# Yersinia Is Not Just the Plague: A Case Report of Yersiniosis Caused by Yersinia enterocolitica

**DOI:** 10.7759/cureus.87381

**Published:** 2025-07-06

**Authors:** Shahnawaz Hashmi, Sunrit Majumder, Zamad Gillani, Mariyam Ali

**Affiliations:** 1 Internal Medicine, South Tees NHS Foundation Trust, Middlesbrough, GBR; 2 Respiratory Medicine, South Tees NHS Foundation Trust, Middlesbrough, GBR

**Keywords:** acute kidney failure, bacterimia, enteric infection, yersinia enterocolitica, yersiniosis

## Abstract

Yersinia enterocolitica is a Gram-negative bacterium that typically causes self-limiting gastrointestinal infections but can sometimes lead to systemic complications, particularly in immunocompromised hosts. This report describes a rare case of a 60-year-old farmer who developed enteric bacteremia, renal impairment, and pulmonary manifestations, likely due to the consumption of undercooked pork. Despite lacking common predisposing factors, the patient presented with a sore throat, joint pain, abdominal discomfort, and a characteristic erythematous rash. Extensive diagnostic workup, including blood cultures and autoimmune screening, yielded negative results. However, stool culture eventually identified Y. enterocolitica as the causative agent. The patient experienced worsening kidney function, respiratory distress, and significant abdominal findings on imaging, prompting consultations across multiple specialties. Treatment with doxycycline and amoxicillin led to clinical improvement, and the patient’s renal function returned to baseline. This case highlights the potential for Y. enterocolitica to cause rare and severe extra-intestinal manifestations, including pulmonary involvement, in an immunocompetent host, underscoring the importance of considering this pathogen in differential diagnoses of systemic infections.

## Introduction

*Yersinia enterocolitica *(*Y. enterocolitica*) is a Gram-negative bacillus belonging to the *Yersinia* genus and the *Enterobacteriaceae* family. It has gained increasing prominence in the last century over its taxonomical counterparts, *Yersinia pestis* and *Yersinia pseudotuberculosis,* as a common cause of gastrointestinal infections [1]. Despite a significant decrease in the number of reported infections attributed to the organism between 2008 and 2016, human yersiniosis is now the fourth most-commonly reported gastrointestinal infection in Europe [2]. 

Generally, *Y. enterocolitica* is a foodborne pathogen that causes infections that are self-limiting and mostly contained within the gastrointestinal tract [3]. However, underlying conditions like malignancy, diabetes, immunosuppression, iron overload, and chronic liver disease can predispose the host to extra-intestinal manifestations [4]. In such instances, the pathogen has a strong propensity to transcend the gastrointestinal barrier and cause systemic complications, resulting in protean manifestations. 

The organism was first described in 1934 under the name *Flavobacterium pseudomallei* after it was isolated by McIver and Pike from the facial abscess of a farmer [5]. Interestingly, the case that we describe here is also of a 60-year-old farmer who in all probability acquired the infection after eating uncooked pork. Despite not having any relevant pre-existing medical conditions commonly associated with yersiniosis, he developed enteric bacteraemia that caused renal impairment and pulmonary manifestations. He presented to us with a sore throat, rash, and arthralgia. Extensive initial investigations failed to reveal a definite cause to sum up his symptoms, until a stool sample grew *Y. enterocolitica.*

## Case presentation

We describe a case of a 60-year-old male farmer who presented to our tertiary care hospital after being referred from a nearby district hospital due to requiring an increased level of support not possible at the district hospital. His symptoms started three days prior to admission, with the patient experiencing sore throat, joint pains and reduced appetite. He reported only taking oral fluids in these three days and noticed he was passing very dark urine. He also had some abdominal pain that was band-like across his abdomen. He denied any changes in his bowel habits, denied any shortness of breath (SOB) or cough or fever. He denied any sick contacts, any history of preceding infection, and any recent travel or hike. He denied any use of alcohol, any recreational drug, any new sexual partners and he had been a non-smoker throughout his life. On further probing, he revealed that prior to the start of these symptoms, he had breakfast in a local eatery where he had pork and beef roast. Vocationally, he works with livestock but denied any recent infections, outbreaks or abortions in his animals. He had no past medical history apart from having well-controlled hypertension and was very fit and well prior to this admission.

On examination, he had fine crackles at the bases of his lung and tenderness in the right lower quadrant. He exhibited widespread joint pain and stiffness, predominantly affecting all the small joints on his hands, hips, ankles, and knees. Additionally, there were slightly raised, erythematous, well-demarcated, blanchable, and painless red spots on his hands, which the patient had not noticed before.

Laboratory tests on admission revealed raised white cell and neutrophil count, high C-reactive protein (CRP), and significant acute kidney injury (Table 1). A CT abdomen and pelvis (CTAP) was done, which showed inflammation of the small bowel mesentery and a short segment of distal ileum, without a clear identifiable cause. Chest X-ray (CXR) showed prominence in both hilar regions but no consolidation was seen. He also had a trans-thoracic echo (TTE) done as the rash on his hands was suspected to be ‘Janeway lesions’; however, no vegetations were visualised on it. 

**Table 1:** Laboratory Data on Admission eGFR: estimated glomerular filtration rate; CRP: C-reactive protein; WBC: white blood cell; Hb: haemoglobin; PLT: platelet; ESR: erythrocyte sedimentation rate; MPOs: myeloperoxidases; PR3: proteinase 3; anti-CCP: anti-cyclic citrullinated peptide; DsDNA: double stranded deoxyribonucleic acid; ENA: extractable nuclear antigen

Investigation	Result	Reference range and units
Sodium	134	133-146 mmol/L
Potassium	4.6	3.5-5.3 mmol/L
Urea	10.3	2.5-7.8 mmol/L
Creatinine	120	50-120 micromol/L
eGFR	56	>90 ml/min
CRP	381	<5.0 mg/L
Lactate	1.9	<2.0 mmol/L
WBC	28.6	4.0-11 x10^9^/L
Hb	154	130-180 g/L
PLT	244	150-400 x10^9^/L
Neutrophils	26.4	2.0-7.5 x10^9^/L
Influenza A/B	negative	
Respiratory syncytial virus	negative	
SARS-CoV-2 virus	negative	
Hepatitis B/C	negative	
ESR	102	2.0-10 mm/1hr
Clostridium difficile	negative	
MPOs	0.26	<3.5 IU/ml
PR3	<0.6	<2.0 IU/ml
Anti-CCP antibodies	0.6	<7.0 U/ml
DsDNA antibody	0.6	<15.0 IU/ml
ENA screen	negative	
C3 complement	1.67	0.75-1.65 g/L
C4 complement	0.36	0.14-0.54 g/L
Rheumatoid factor	<10.3	<20 IU/ml
Antinuclear factor antibody	negative	

Given the acute history and wide array of symptoms, the patient consulted with a microbiologist and was started on doxycycline and gentamicin to cover for any atypical infection. Test results were sent off for infections like leptospirosis, *Coxiella*, *Brucella*, *Borrelia*, and *Listeria*. The patient had five negative blood cultures so far and the autoimmune screen was negative as well. Patient's kidney function kept on worsening and there was no improvement seen in the joint pain so rheumatology and nephrology were taken on board as well along with an infectious disease (ID) team. The patient’s oxygen requirement increased significantly to 40% venturi and he was moved to the respiratory ward.

**Figure 1:**
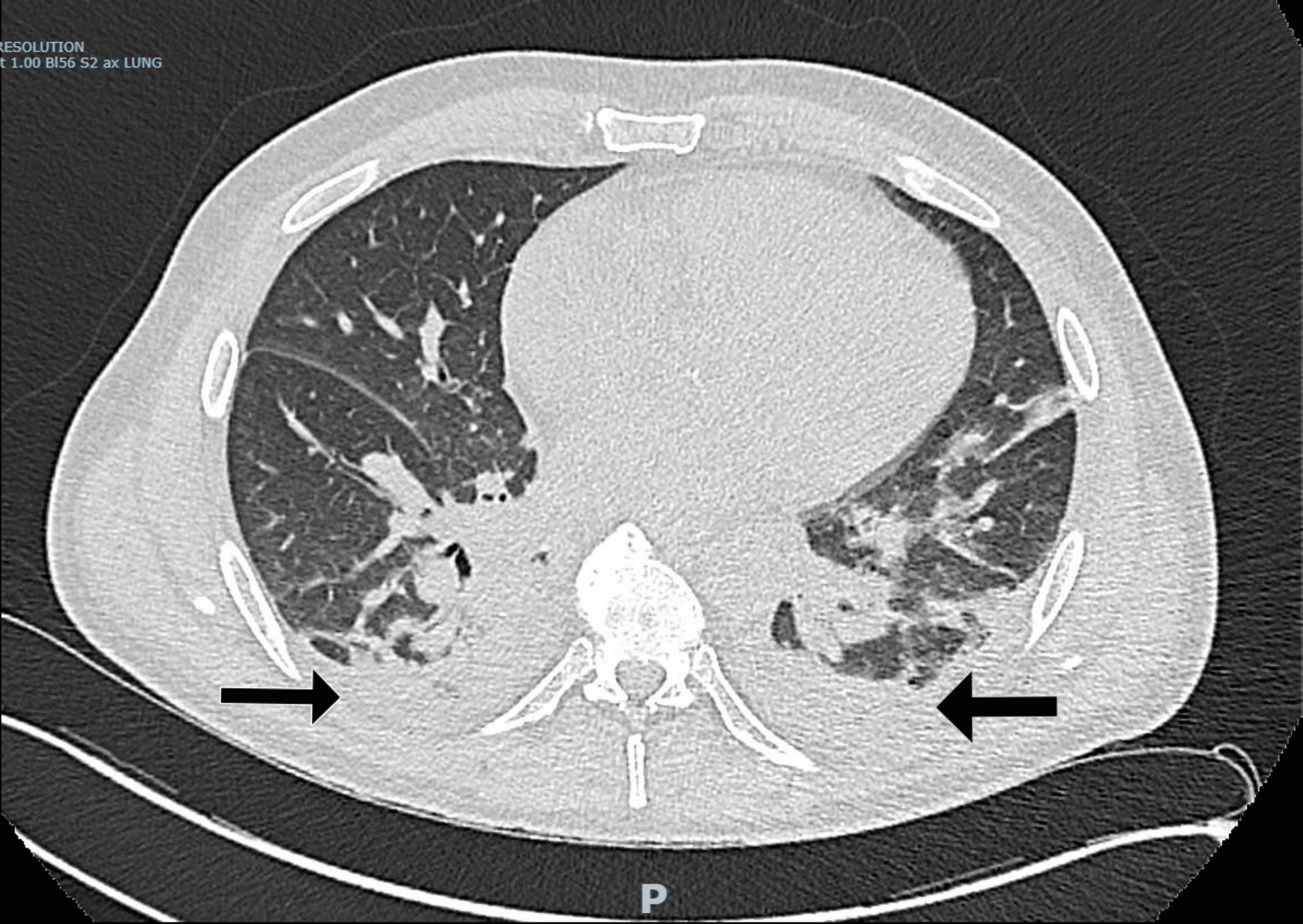
CT showing peribronchovascular opacification and bilateral pleural effusions. Note the arrows pointing towards effusions and opacifications

**Figure 2:**
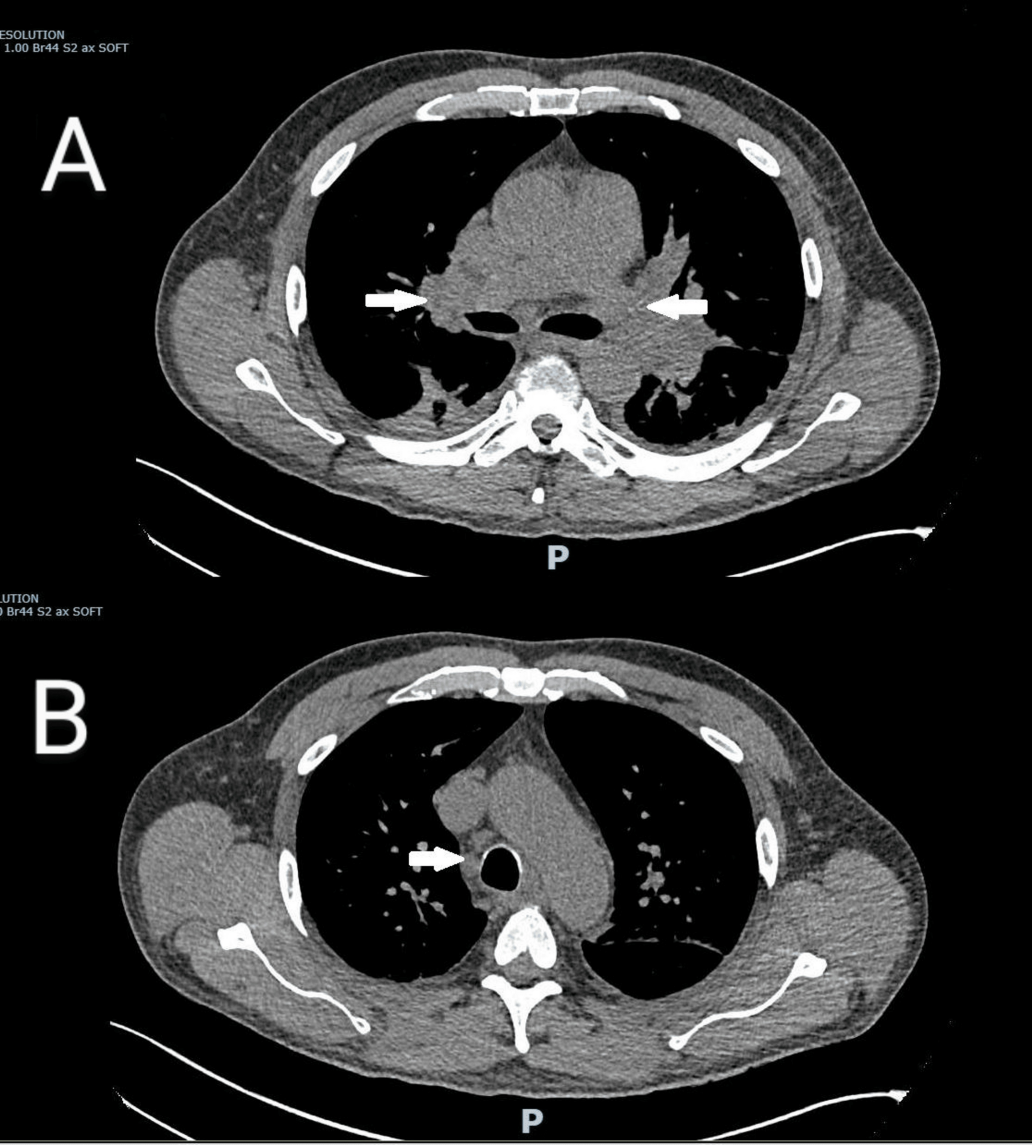
CT showing extensive bilateral hilar and mediastinal lymph nodes Note the arrows pointing to the enlarged lymph nodes in panels A and B

The rheumatology team, after reviewing the patient, thought the condition to be infective rather than any autoimmune condition, considering all the autoimmune tests were negative, and hence did not start any steroids or other medications. However, considering the ongoing pain in the hip joint and likely infective picture of the patient, they requested an MRI hip and lumbosacral spine to rule out any bony involvement, which showed no evidence of spondylodiscitis or septic hip arthritis. The nephrology team also reviewed the patient and they thought the condition to likely be infective glomerulonephritis. However, they advised to send anti-streptolysin O (ASO) titres, autoantibodies (anti-glomerular basement membrane (GBM)) and to continue with intravenous (IV) hydration as patients’ urine output had started to increase. So far, the differential diagnosis list was quite broad, but the main suspicion was that this was an infective pathology, considering we had ruled out various autoimmune conditions like vasculitis or sarcoidosis. Because of the ongoing oxygen requirement, the patient had a high-resolution CT scan (HRCT) of his chest which showed new extensive bilateral hilar and mediastinal lymph nodes, peribronchovascular opacification and bilateral pleural effusions (Figures 1, 2).

The ID team recommended sending Hantavirus serology, *Legionella* and *Mycoplasma* screen, and changed the gentamicin to amoxicillin and continued with doxycycline. With the antibiotics change and ongoing fluid therapy, the patient's condition started improving a bit and oxygen requirement decreased to 4L via nasal cannula. ASO titres came back as negative, and repeat blood cultures were again negative. However, there was not much improvement seen in renal function tests(Table 2)*.* 

**Table 2:** Repeat investigations and blood work eGFR: estimated glomerular filtration rate; GBM: glomerular basement membrane; IgG: immunoglobulin G; IgM: immunoglobulin M; IgA: immunoglobulin A; HIV: human immunodeficiency virus; DNA: deoxyribonucleic acid; ACE: angiotensin converting enzyme

Investigations	Results	Reference range and units
Sodium	122	133 – 146 mmol/L
Potassium	5.3	3.5 – 5.3 mmol/L
Urea	41.2	2.5 – 7.8 mmol/L
Creatinine	505	50 – 120 micromol/L
eGFR	10	> 90 ml/min
GBM antibodies	<1.5	<7.0 U/ml
Borellia burgdorferi IgG/IgM, Brucella IgG/IgM	Not detected	
Crytosporidium/Giardia/Campylobacter/Salmonella	Not detected	
Shigella/shiga toxin	Not detected	
Coxiella burnetti IgG/IgM	Not detected	
Mycoplasma IgM	Not detected	
Endemic typhus IgG/IgM	negative	
Spotted fever IgG/IgM	negative	
HIV 1/2 antigen/antibody	Not detected	
Tropheryma whipplei DNA, Rickettsia DNA	Not detected	
Serum ACE	<20	20 – 70 U/L
Antistreptolysin O antibody	27	< 200 IU/mls
Legionella antigen	Not detected	
Chlamydia pneumoniae	Not detected	
Leptospira interrogans DNA/IgM antibody	Negative/not detected	
Immunoglobulin G	6.86	6.0 – 16.0 g/L
Immunoglobulin A	3.51	0.8 – 4.0 g/L
Immunoglobulin M	0.69	0.5 – 2.0 g/L

The patient was reviewed by the nephrology team again and rate of fluid therapy was adjusted and the patient was booked for an ultrasound of the renal tract (USS KUB). Tests were repeated and showed improvement in the renal function and the USS did not show any renal pathology. *Legionella*, *Borrelia*, *Rickettsia*, *Brucella*, *Coxiella*, *Tropheryma whipplei*, hepatitis C, HIV, and autoimmune screen all came back as negative. 

Patient developed new onset diarrhoea in his second week of admission and had an outbreak of blanching, serpiginous rash on his chest, abdomen and thighs. Stool sample was sent for culture and dermatology input was sought regarding the rash, whose impression was that the rash was most likely reactive to the ongoing internal process. 

**Figure 3:**
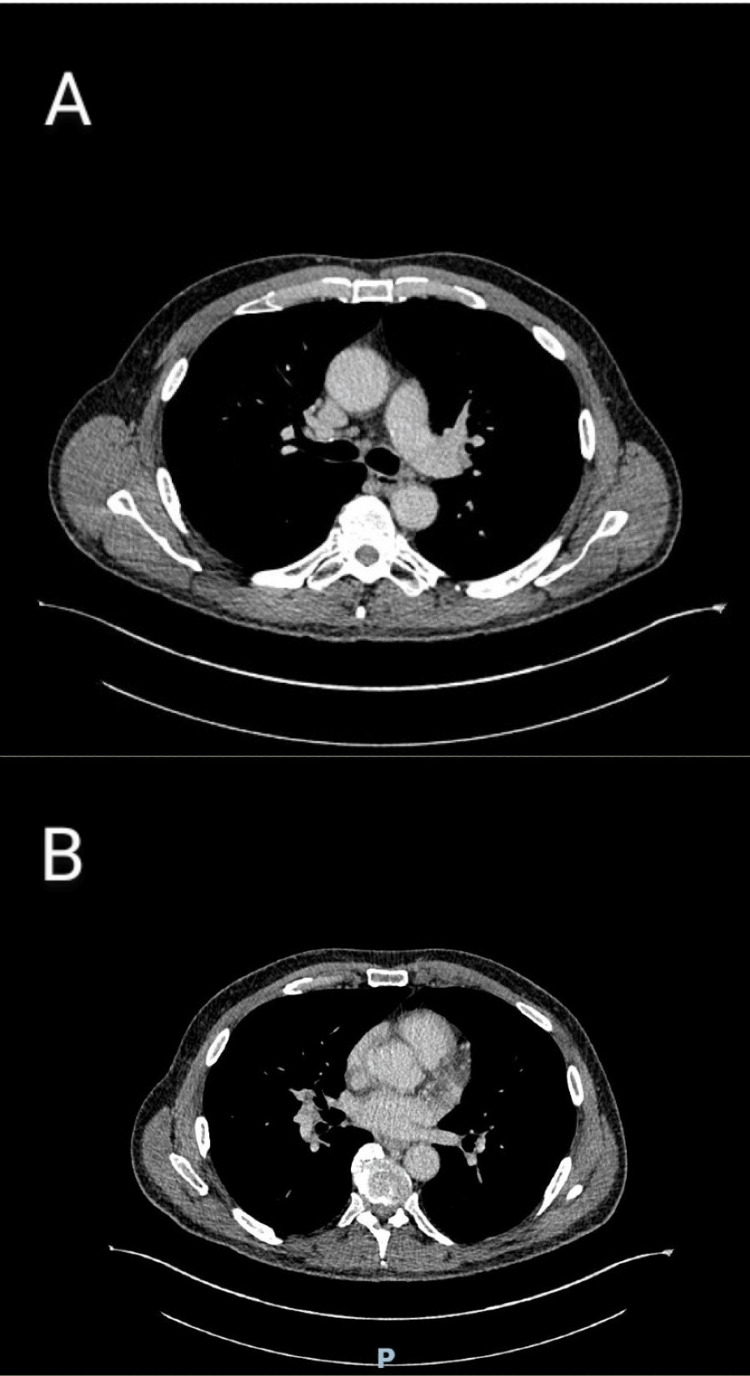
Outpatient CT scan showing resolution of the hilar and mediastinal lymphadenopathy Comparing this image with the previous images, you can see that the panel A shows the hilar region without the enlarged lymph nodes seen in Figure 2 while panel B shows the mediastinal region without the previously seen enlarged lymph nodes in Figure 2.

Patient kept on improving with ongoing therapy, was weaned off oxygen completely and his kidney functions went to baseline. The stool sample that was sent at the onset of diarrhoea grew *Y. enterocolitica*, which explained the clinical picture so far, as this organism can cause yersiniosis. Due to the concern regarding food poisoning, the United Kingdom Health Security Agency (UKHSA) was contacted about the diagnosis after informing this to the patient as well. The patient improved clinically and was discharged home with oral doxycycline and outpatient (OP) follow-up. A subsequent OP CT chest showed resolution of the lung inflammatory changes and significant reduction in volume of mediastinal and hilar nodes (Figure 3), confirming the cause of them to be infective.

## Discussion

*Y. enterocolitica* is primarily a gastrointestinal pathogen, but at times can traverse the gastrointestinal barrier to produce extra-intestinal manifestations, especially in hosts with predisposing factors. Bacteraemia is particularly prominent in the presence of immunosuppression, in recipients of blood transfusion, or in patients with iron overload [3,4]. The absence of any of these underlying risk factors in an otherwise immunocompetent host makes this case a rare presentation. The most prevalent route of acquisition of the pathogen is through contaminated food and water (especially uncooked pork or unpasteurised milk) or through blood transfusion. The biotype most commonly responsible for human infections globally is biotype 4 and pigs are the main reservoir for it [3,6]. Although they are asymptomatic carriers of the organism, contamination of pork meat can occur during slaughter. This fits with the history of consuming a roast meal of pork prior to the onset of our patient’s symptoms. Apart from that, he did not have any relevant history of prior blood transfusions or recent travel, and, although he worked as a livestock farmer, he had no pigs on his farm. 

Following entry into the intestinal lumen, the organism must colonise the intestinal tract as a starting point in establishing its pathogenicity. It does so with the help of a 64-75 kb plasmid, expressed only in the virulent forms, which codes for a protein cascade that helps to guide the organism through the layers of the intestine and finally the mucosal layer overlying the brush border epithelium [7]. The organism then synthesizes its large outer membrane protein (YadA), which aids its binding to the brush border epithelium, especially in the terminal ileum and proximal colon, where the majority of its intestinal manifestations like terminal ileitis, mesenteric adenitis, and pseudoappendicitis are seen [8]. In the case that we have discussed, a CT abdomen-pelvis done during the initial course of the disease had revealed inflammation of the distal ileum and the associated mesentery. For most patients, the infection is restricted to the gastrointestinal tract; however, in some cases like the one presented in this report, the pathogen spreads beyond to cause protean manifestations. These include pneumonia, empyema, septic arthritis, dermatological manifestations including erythema nodosum, pustules, and vesicobullous lesions, liver and splenic abscesses, meningitis, osteomyelitis, endocarditis, glomerulonephritis, myocarditis, and mycotic aneurysms [3,9]. Although arthralgia is supposed to result from an immune response to *Yersinia,* the lung abscess results from direct invasion of bacteria. What was particularly interesting in our patient were the lung manifestations - while pulmonary manifestations like pneumonia and nodular infiltrates have been reported with yersiniosis, hilar lymphadenopathy as a manifestation has rarely been mentioned in case reports [10]. The recognition of the same makes the disease a rare differential in patients presenting with similar signs and symptoms. 

*Y. enterocolitica* is generally a self-limiting infection, but treatment is warranted in moderate to severe infections. Although definitive guidelines for the management of the infection do not exist, it has been reported that the organism is susceptible to most antibiotics except beta-lactams including penicillin, ampicillin, and first-generation cephalosporins [11]. While it is difficult to accurately predict which antibiotic the pathogen in this case was susceptible to, combination therapy with doxycycline and amoxicillin led to discernible improvements in clinical condition. Although the efficacy of amoxicillin alone in treating the infection is debatable, most published literature has highlighted the susceptibility of the *Y. enterocolitica* isolates to doxycycline [11]. 

## Conclusions

This case highlights the potential for *Y. enterocolitica* to cause severe, extra-intestinal manifestations such as bacteremia, renal impairment, and pulmonary complications, even in immunocompetent individuals. It underscores the importance of considering this pathogen in the differential diagnosis of systemic infections, particularly in patients with gastrointestinal symptoms and a history of consuming undercooked pork. Early identification and targeted treatment are crucial for improving outcomes in such rare and complex cases and we hope this case report increases clinical awareness about yersiniosis. 
